# Zinc-Based bioMOF Nanocarrier for Curcumin Release Enables Permeation through Biomimetic Biological Membranes and Controlled Release

**DOI:** 10.1021/acsomega.6c00840

**Published:** 2026-07-17

**Authors:** Lorrayne Ohana Coelho, Marcos Vinicius de Sousa Pereira, Tatianny de Araujo Andrade, Katia M. Oliveira, Jemmyson R. de Jesus

**Affiliations:** † Research Laboratory in Bionanomaterials, LPbio, Department of Chemistry, Federal University of Viçosa, 36570-900 Viçosa, Minas Gerais, Brazil; ‡ National Institute of Science and Technology of Bioanalytics-Lauro Kubota (INCTBio-LK), 13083970 Campinas, SP, Brazil; § 28127 University of Brasilia, Institute of Chemistry, CEP 70910-000 Brasília-DF, Brazil

## Abstract

Curcumin (Cur) is a natural polyphenolic compound with significant therapeutic potential, including antioxidant and anti-inflammatory activities. However, its clinical application is limited by its low aqueous solubility and low bioavailability. In this study, we developed a biocompatible Zn-based metal–organic framework (bio-MOF) as a nanocarrier for curcumin. The synthesized material was characterized by powder X-ray diffraction (P-XRD), fourier transform infrared spectroscopy (FT-IR), scanning electron microscopy with energy dispersive X-ray spectroscopy (SEM-EDS), and thermogravimetric analysis (TG/dTG), confirming the stability of the structure and the successful loading of the drug. The system showed a Cur loading efficiency of 75.98 ± 2.65% (*n* = 3). Cytotoxicity assays were performed in MCF-7 cell lines and demonstrated that the delivery system has a superior effect on reducing cell viability compared to free cur. The release kinetics followed a Ritger-Peppas model (*R* ^2^ = 0.948), indicating controlled release behavior. *In vitro* permeation studies revealed that the carrier successfully penetrated the biomimetic barrier and released Cur in the receptor compartment. Finally, through albumin fluorescence quenching analysis, a progressive decrease in fluorescence was observed with a Stern–Volmer constant (*K*_SV_) of 9.4 × 10^3^ M^–1^, indicating a moderate binding affinity and suggesting that the nanocarrier can effectively interact with transport proteins. These findings demonstrate that the bio-MOF platform is a promising strategy to enhance the release and biological interaction of hydrophobic phytochemicals.

## Graphical abstract

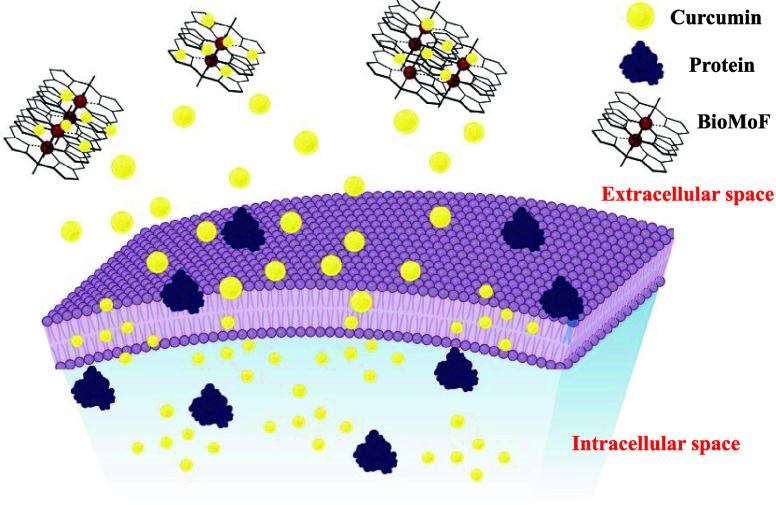

## 1 Introduction

Humans have utilized plants to treat diseases or alleviate symptoms for more than 30,000 years.^1,2^ Over time, this practice has been consolidated through empirical knowledge and accidental discoveries.^3^ Currently, the vast diversity of species and their therapeutic properties has prompted modern medicine to investigate the potential effects of various plant extracts.^4^ Their biological affinity and the lower incidence of adverse effects compared to synthetic drugs make phytopharmaceuticals promising targets for researchers worldwide.^5–7^

Curcumin (Cur) is a natural polyphenolic compound derived from turmeric (*Curcuma longa* *L*.), widely recognized for its extensive use in traditional Asian medicine as well as its role as a natural additive in culinary applications.^8^ In addition to its commercial value in the food and cosmetic industries, it attracts significant interest within the pharmaceutical sector.^9^ Cur exhibits antioxidant, anti-inflammatory,^10–12^ anticancer,^13^ cardiovascular,^14^ neuroprotective, and antimicrobial activities.^8,15^ These effects stem from key interactions between Cur and transcription factors, proteins, cytokines, and inflammatory mediators, promoting its therapeutic potential.^8,16^

Recent studies demonstrate that Cur acts as a chemosensitizer, reducing the overexpression of the multidrug resistance complex (MDR-1) and aldehyde dehydrogenase-1 (ALDH-1), resulting in a decrease in the population of cancerous stem cells. Furthermore, Cur potentiates the apoptotic effect of the chemotherapeutic agent paclitaxel by reducing caspase activation, providing a synergistic cytotoxic interaction in MCF-7 cells and reducing tumor burden *in vivo*.^17^

Despite its broad range of biological activities, the clinical administration of free Cur faces significant limitations associated with its unfavorable pharmacokinetic properties. Its hydrophobic nature leads to poor aqueous solubility (0.6 μg mL^–1^) and low absorption in the gastrointestinal tract.^18^ Furthermore, its rapid systemic metabolism and limited permeability across biological barriers diminish its therapeutic potential.^8,19^ In this context, nanotechnology has been investigated through various nanosystems as a strategy to circumvent the limitations of active substances.^20,21^ Several formulations based on hydrogels,^22–24^ emulsions^25–27^ and nanocapsules^28–31^ have been employed to overcome these challenges.

Liu et al. developed hydrogel microspheres in which curcumin was encapsulated via emulsions stabilized by gelatin, cellulose nanocrystals, and sodium alginate.^31^ The resulting material exhibited an encapsulation efficiency exceeding 90%, gastrointestinal pH sensitivity, and a controlled release profile capable of prolonging Cur retention in this region. In another study, Teixé-Roig et al.^32^ evaluated the pharmacokinetics and biodistribution of Cur administered orally to rats using optimized emulsions formulated with 50% medium-chain triglycerides and soy lecithin as a plant-based emulsifier, with measurements performed at 2 and 4 h postadministration.^33^ Total bioavailability of curcuminoids increased by a factor of 10.6 when rats were administered the curcumin emulsion compared to the control suspension.

Metal–organic frameworks (MOFs) represent another class of materials with compelling properties for the protection of bioactive compounds. Their porous nature allows for tunable synthesis through the coordination of metal ions and organic ligands.^34,35^ Notable characteristics of MOFs include high thermal stability, large surface area, and tailorable pore sizes, which render them versatile for biomedical applications.^36–38^ The incorporation of therapeutic agents into MOFs yields materials with enhanced biological activity.^38–40^

Aiming to enhance the therapeutic potential of curcumin (Cur), this study focuses on developing a delivery platform based on a biocompatible MOF (bio-MOF). The synthesis, following a mixed-ligand approach, utilizes Zinc (Zn), benzene-1,3,5-tricarboxylic acid (Btc­(COOH)_3_), and the amino acid l-phenylalanine (Phe). This strategy aims to create a porous matrix capable of protecting Cur from premature degradation while ensuring low systemic toxicity and conferring biorecognition to the carrier.

## 2 Materials and Methods

### 2.1 Materials

Lyophilized bovine serum albumin (BSA) and benzene-1,3,5-tricarboxylic acid (Btc­(COOH)_3_) were acquired from Sigma-Aldrich (SP, Brazil). l-phenylalanine (Phe) was supplied by ACS Científica (SP, Brazil). Sodium chloride (NaCl) was obtained from Impex (SP, Brazil). Potassium chloride (KCl) and zinc nitrate (Zn­(NO_3_)_2_) supplied by Vetec (RJ, Brazil). Dimethyl sulfoxide (DMSO) was provided by Neon Comercial Ltd.a (SP, Brazil). Ethylene glycol was obtained from Nova Cinética Ind e Com de Prods Químicos Ltd.a (SP, Brazil). Dibasic potassium phosphate (K_2_HPO_4_) was obtained from Labsynth (SP, Brazil). Sodium dodecyl sulfate (SDS) and dibasic sodium phosphate (Na_2_HPO_4_·7H_2_O) were supplied by Dinâmica Química (SP, Brazil). Ethyl alcohol (C_2_H_6_O) was obtained from Êxodo Científica (SP, Brazil). Cur was obtained from Spectrum Chemical (CA, USA). All reagents were of analytical grade and used as received without further purification. Solutions were prepared using deionized water from a Milli-Q system (Millipore, Bedford, MA, USA), with resistivity exceeding 18.0 MΩ·cm.

### 2.2 Synthesis [Zn­(Btc­(COO)_3_)­(Phe)]*_n_*

The [Zn­(Btc­(COO)_3_)­(Phe)]*_n_* was prepared via a solvothermal method, following a procedure adapted from Jesus et al.^33^ Initially, 0.8 g of 1,3,5-benzenetricarboxylic acid (Btc­(COOH)_3_) and 0.1 g of l-phenylalanine (Phe) were dissolved in 8.0 mL of deionized water, after which 500 μL of NaOH solution (8 mol L^–1^) was added to promote ligand deprotonation. Subsequently, 0.6 g of Zn­(NO_3_)_2_ was incorporated into the solution, under magnetic stirring for 5 min. Sequentially, ethylene glycol (4.5 mL) were then added as a cosolvent, and the resulting mixture was transferred to a Teflon-lined reactor, sealed in a stainless-steel autoclave, and heated at 200 °C for 24 h. Upon completion of the reaction, the resulting solid product was isolated by centrifugation at 4000 rpm for 20 min, followed by washing with a hydroalcoholic solution (ethanol/water, 50% v/v) to remove residual reactants and byproducts. The purified material was subsequently dried in an oven at 60 °C for 10 h. Figure 1 illustrates the preparation of the bioMOF and the experimental studies performed.

**Figure 1:**
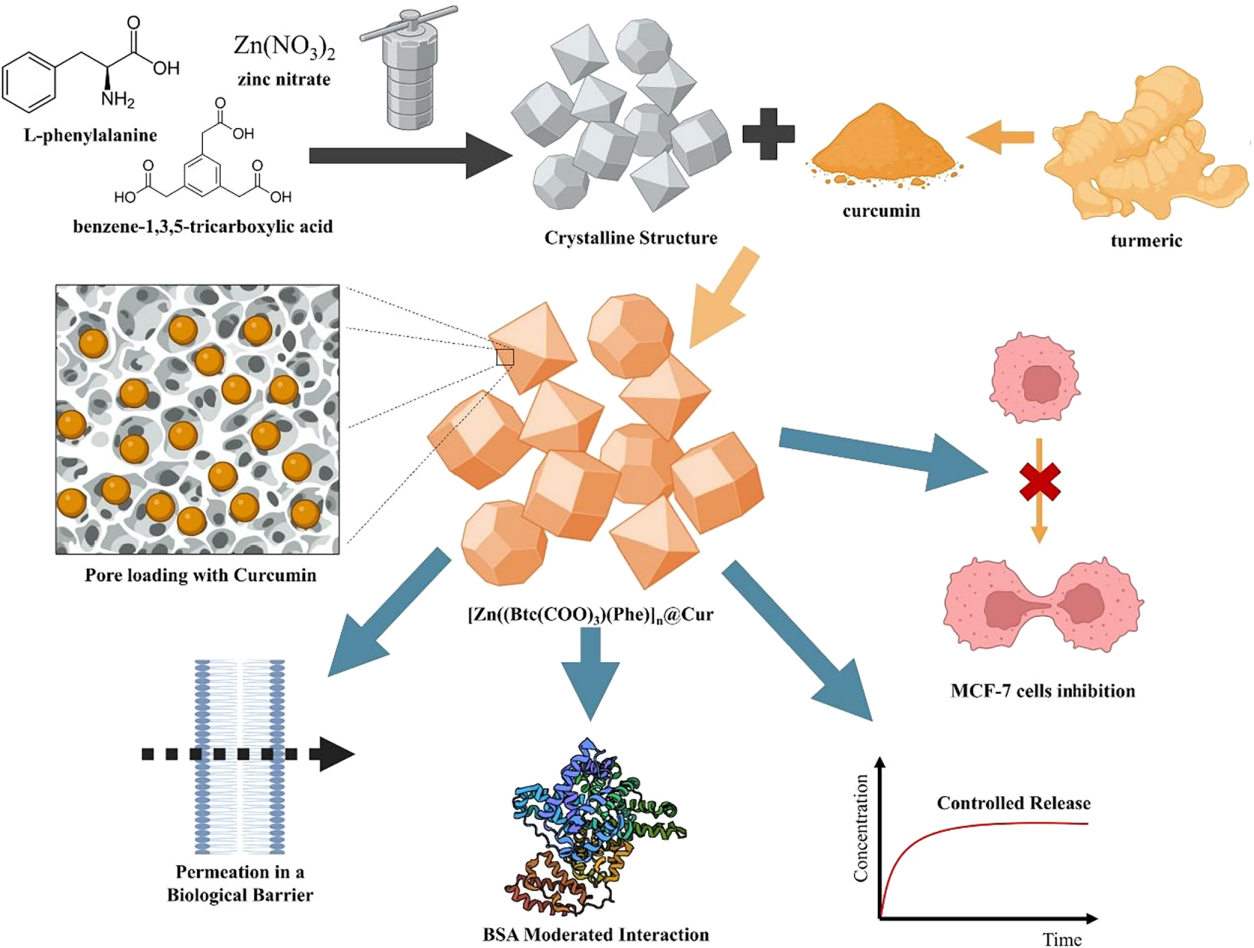
Schematic illustration of bioMOF preparation and the studies performed. Abbreviations: Btc­(COO)_3_, benzene-1,3,5-tricarboxylic acid; Phe, l-phenylalanine; Cur, curcumin. Created with BioRender.com.

### 2.3 Characterization

The structural and physicochemical properties of the materials were systematically characterized using complementary analytical techniques.

The crystalline structure and phase purity were evaluated by powder X-ray diffraction (P-XRD) at room temperature using a Rigaku diffractometer (Tokyo, Japan) operating in Bragg–Brentano geometry, with data collected in the 2θ range of 25–70° employing Cu Kα radiation. Morphological features and elemental composition were investigated by scanning electron microscopy coupled with energy-dispersive X-ray spectroscopy (SEM-EDS), using a JEOL JSM-360-LV microscope (Tokyo, Japan) operated at 20 kV in secondary electron mode. Prior to analysis, the samples were mounted on a Cu–Zn holder and coated with a thin gold layer (∼10 nm) using a sputter coater (Bal-tec MED020, Balzers, Liechtenstein) to improve conductivity.

Functional group identification was carried out by Fourier transform infrared spectroscopy (FT-IR) using a spectrophotometer from Varian (CA, USA), equipped with an attenuated total reflectance (ATR) accessory and a germanium crystal, operating in the mid-infrared region (400–4000 cm^–1^). Thermal stability was evaluated by thermogravimetric analysis (TG/dTG) using a thermobalance from PerkinElmer (MA, USA). Approximately 3.0 mg of sample were heated from 25 to 900 °C at a rate of 10 °C min^–1^ under a nitrogen flow of 50.0 mL min^–1^. In addition, the surface area and pore size distribution were determined using a surface area analyzer from Anton Paar (Graz, Austria). The Brunauer–Emmett–Teller (BET) and t-plot models were applied to nitrogen adsorption–desorption isotherms measured at 77 K over a relative pressure range of 0.0001 ≤ *p*/*p*_0_ ≤ 0.9918. Rouquerol’s criteria were applied to evaluate a physically significant (positive) C constant.

### 2.4 Loading Efficiency

Considering that MOFs are porous materials with a high capacity for encapsulating active compounds,^41^ the loading of curcumin in [Zn­(Btc­(COO)_3_)­(Phe)]*_n_* carried out following a procedure adapted from Wu et al. (2025),^42^ with minor modifications. Initially, a Cur stock solution was prepared (1 mg mL^–1^) in DMSO, and 50 mg of [Zn­(Btc­(COO)_3_)­(Phe)]*_n_* was dispersed in 10 mL of this solution for a period of 24 h. After this time, the suspension was centrifuged at 4000 rpm for 25 min, and the supernatant was then carefully removed. The concentration of nonencapsulated curcumin in the supernatant was determined by UV–vis spectrophotometry at λ_max_ = 426 nm. The amount of Cur loaded into the MOF structure [Zn­((Btc­(COO)_3_)­(Phe)]*_n_* @Cur) was estimated by calculating the difference between the initial and residual concentrations in solution, as described by eq 1.

1 $$E_{f}=\left(\frac{C_{i}-C_{f}}{C_{i}}\right)\times 100$$

Where *E*_f_ represents the loading efficiency, *C*_i_ and *C*_f_ the initial and final Cur concentrations, respectively.

### 2.5 Cell Viability

Human breast adenocarcinoma cells (MCF-7, ATCC no. HTB-22) were used in this study. The cells were cultured in RPMI-1640 medium supplemented with 10% fetal bovine serum, 100 U mL^–1^ penicillin, 100 μg mL^–1^ streptomycin, and 2 mM L^–^glutamine, and maintained at 37 °C in a humidified atmosphere containing 5% CO_2_.^43^

Cell viability was assessed using the MTT assay. Briefly, cells were seeded in 96-well plates at a density of 1.5 × 10^4^ cells per well in 150 μL of complete medium and incubated for 24 h to allow cell adhesion. The test compounds were dissolved in DMSO and added to the wells in a volume of 0.75 μL, resulting in a final DMSO concentration of 0.5% (v/v). Cells treated with 0.5% DMSO alone were used as the negative control. After treatment, the cells were incubated for 48 h under the same conditions. Subsequently, 50 μL of MTT solution (0.6 mg mL^–1^ in PBS) was added to each well, followed by an additional 4 h incubation to enable the reduction of MTT to formazan by metabolically active cells.^44^ The resulting formazan crystals were then dissolved in 150 μL of DMSO per well, and absorbance was measured at 570 nm using a Varioskan LUX Multimode Microplate Reader. Cell viability was determined based on the extent of MTT reduction.

### 2.6 Release Kinetics

The kinetic study was conducted based on a methodology adapted from Rebekah et al.^44^ In short, 150 mg of [Zn­(Btc­(COO)_3_)­(Phe)]*_n_* @Cur and 1 mL PBS (pH = 7.4) were sealed in a cellulose dialysis membrane. The system was immersed in 30 mL of PBS buffer solution and was kept under constant stirring and temperature of 37 °C.^45^ At predefined intervals, the apparent concentration of curcumin was obtained in a UV–vis spectrophotometer (λ_max_ = 426 nm), and quantified through an analytical curve. To understand the release mechanism, the data were fitted to Higuchi (eq 2) and Ritger-Peppas (eq 3) models.^46^

2 $$q_{t}=K_{H}\cdot t^{1/2}$$

3 $$\frac{q_{t}}{q_{e}}=K\cdot t^{n}$$

Where *q_t_* refers to the amount of Cur released (mg mg ^–1^) at a given time *t* (min), while *q*_e_ correspond to the amount of Cur released at equilibrium. *K*_H_ is the Higuchi dissolution constant, *K* is the release rate constant from the Ritger-Peppas model. The parameter *n* is the release exponent, which provides information about the drug release mechanism. The *q*_e_ values were determined using eq 4

4 $$q_{e}=\frac{{(C}_{i}-C_{e})\times V}{m}$$

*C*_i_ and *C*_e_ are the initial and equilibrium concentration (mg L^–1^), respectively. *V* represents the solution volume (L), and *m* is the mass of the nanocarrier (mg).

### 2.7 Permeation in Biological Barrier

The permeation across biological barriers was evaluated using Franz-type diffusion cells coupled with porcine ear skin as a biomimetic membrane model (*n* = 3).^47^ Briefly, the skin was properly prepared, shaped, and secured in the diffusion cell. A suspension of [Zn­(Btc­(COO)_3_)­(Phe)]*_n_* @Cur (97 mg L^–1^) was applied to the membrane. PBS buffer (pH = 7.4) was used as the receptor solution, and the permeation assay was carried out at 37 ± 0.5 °C for 24 h under controlled conditions. After the permeation period, the different skin layers were isolated to evaluate retention of Cur. The stratum corneum was removed using the tape-stripping technique, while the epidermis was separated after immersion of the skin in deionized water at 60 °C for 45 s, followed by gentle scraping with a scalpel. The remaining dermal layer was fragmented into small sections. Each isolated layer was transferred to tubes containing acetonitrile, vortex-mixed for 2 min, and subsequently sonicated for 30 min to extract the retained Cur. The extracted solutions were analyzed by UV/vis spectrophotometry at λ_max_ = 426 nm for quantification.^48^

### 2.8 Study of Interaction with Albumin

The study of interaction between BSA and [Zn­(Btc­(COO)_3_)­(Phe)]*_n_* @Cur was conducted following the methodology described by Moreira et al.^46^ The analysis was based on the intrinsic fluorescence of tryptophan residues. Fluorescence spectra were obtained with an Avantes AvaspecHS2048XL fluorimeter (Apeldoorn, The Netherlands). First, the emission spectrum of a BSA solution (1.0 μmol L^–1^, 2.0 mL) was recorded Subsequently, aliquots of a suspension containing [Zn­((Btc­(COO)_3_)­(Phe))]*_n_* @Cur were progressively added to the protein solution, resulting in final biomaterial concentrations ranging from 0 to 4.0 μmol L^–1^. After each addition, fluorescence spectra were collected, and changes in emission intensity were monitored to assess the interaction between BSA and the bioMOF system. The fluorescence quenching behavior was quantitatively evaluated using the Stern–Volmer model, as described by eq 5

5 $$\frac{I_{0}}{I}=1+K_{\mathrm{SV}}\left[Q\right]$$

In this equation, *I*_0_ and *I* are the fluorescence intensities of the protein in the absence and presence of the quencher, respectively, while [Q] represents the quencher concentration, and *K*_SV_ is the Stern–Volmer quenching constant. The interaction between the biomaterial and BSA was evaluated by constructing a linear plot of *I*_0_/*I* versus [Q] allowing the determination of the quenching constant and assessment of the fluorescence suppression process.

### 2.9 Statistical Analysis

Statistical analyses were carried out using Origin software (version 2018). Significant differences among experimental groups were evaluated by analysis of variance (ANOVA) followed by Tukey’s test. All experiments were conducted in triplicate, and the results were expressed accordingly.

## 3 Results and Discussion

### 3.1 Characterization

X-ray diffraction analyses were conducted for both the as-synthesized and curcumin-loaded materials to evaluate their crystallinity and verify the preservation of the structural framework after the loading process. The resulting diffractograms are shown in Figure 2A. Both samples exhibit sharp and intense diffraction peaks, confirming the crystalline nature of the original structure and its stability after the loading process.^49^

**Figure 2:**
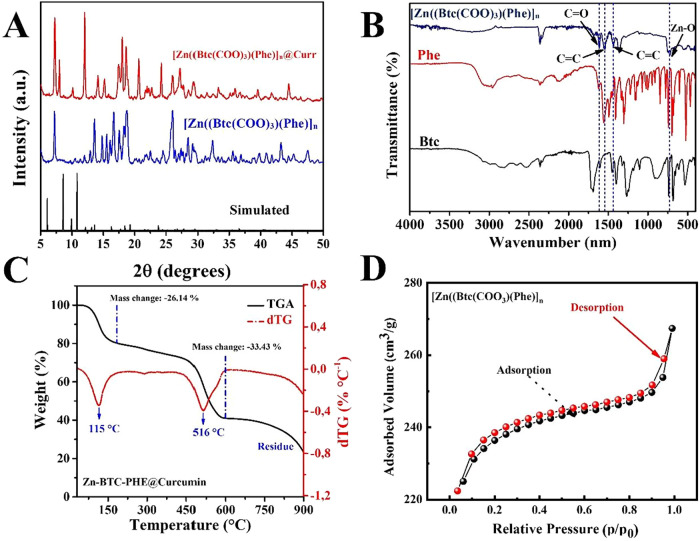
Results of bioMOF characterization. (A) Powder X-ray diffraction (P-XRD) pattern of the pure material [Zn­(Btc­(COO)_3_)­(Phe)]*_n_*, of loaded material [Zn­(Btc­(COO)_3_)­(Phe)]*_n_* @Cur and of the simulated pattern (CCDC 279674).^50^ The observed peaks indicate the presence of characteristic crystalline phases, allowing the identification and evaluation of the compound’s crystalline phases. (B) Fourier Transform Infrared (FT-IR) Spectra for [Zn­(Btc­(COO)_3_)­(Phe)]*_n_* and the ligands 1,3,5-benzenetricarboxylic acid (Btc) and l-phenylalanine (Phe). (C) Thermogravimetric analysis (TG/dTG) curves of [Zn­(Btc­(COO)_3_)­(Phe)]*_n_* @Cur. (D) N_2_ adsorption/desorption curve at 77 K and specific surface area (BET), indicating the porous nature of the material.

The occurrence of intense reflections at low 2θ angles is evidence of the significant porosity of the material.^51,52^ Consequently, the decrease in peak intensity below 20° in the loaded sample suggests that Cur is occupying the pores of the crystal lattice [Zn­(Btc­(COO)_3_)­(Phe)]*_n_*. Comparison with the simulated standard (CCDC 279674)^50^ demonstrates that the addition of the Phe ligand promotes structural modifications in relation to the Zn-Btc MOF, with the emergence of peaks at 2θ angles greater than 15°.

Figure 2B presents the FTIR spectra of the materials Btc, Phe and the material [Zn­(Btc­(COO)_3_)­(Phe)]*_n_*. The preservation of some characteristic bands of the precursors is noted, but with shifts and changes in intensity, indicating coordination between Zn^2+^ and the organic ligands. The decrease in the intensity of the C=O band and the emergence of new bands in the region of 500–700 cm^–1^ suggest the formation of metal–oxygen (Zn–O) bonds, confirming the synthesis of the hybrid material.

Figure 2C shows the TG/dTG analysis of the material. An initial mass loss is observed around 100 °C, attributed to the elimination of physically adsorbed water molecules. Then, a second degradation occurs between approximately 200 and 400 °C, related to the decomposition of the organic components (Btc and Phe) bound to the structure. After 400 °C, the rate of mass loss decreases considerably, suggesting the formation of more stable residues, possibly metal oxides resulting from the decomposition of the [Zn­(Btc­(COO)_3_)­(Phe)]*_n_*. The thermal profile obtained demonstrates the thermal stability of the compound and highlights the degradation stages of the organic constituents, which are essential for applications that require moderate thermal resistance.

Figure 2D presents the N_2_ adsorption/desorption isotherm of the [Zn­(Btc­(COO)_3_)­(Phe)]*_n_* material measured at 77 K. The isotherm displays a steep uptake at low relative pressures (*P*/*P*_0_ < 0.1), which is characteristic of a type I isotherm according to the IUPAC classification, indicating the predominance of a microporous framework. In addition, the absence of a significant hysteresis loop suggests a negligible contribution of mesoporosity to the overall pore structure. The specific surface area was initially estimated using the BET model, yielding a value of approximately 711.5 m^2^ g^–1^. However, the calculated BET C constant was negative, indicating the absence of a physically meaningful BET region. Consequently, the adsorption data were re-evaluated according to the Rouquerol consistency criteria, which confirmed that no appropriate pressure range yielded a positive C constant, demonstrating that the BET model is not suitable for accurately describing this adsorption system. This behavior is commonly observed in highly microporous materials, where adsorption at low relative pressures is predominantly governed by micropore filling rather than multilayer adsorption. To achieve a more reliable textural characterization, the t-plot method was employed (Figure S1). The analysis revealed a micropore volume of approximately 0.41 cm^3^ g^–1^ and a relatively low external surface area (∼20 m^2^ g^–1^), further confirming the strongly microporous nature of the material. The significant internal porosity of [Zn­((Btc­(COO)_3_)­(Phe))]*_n_* suggests its potential applicability in processes involving solid–molecule interactions, including encapsulation and controlled release systems.

The SEM images obtained are presented in Figure 3A,B. It can be observed that the sample presents particles with predominantly irregular morphology, with the presence of well-defined lamellar structures and smaller agglomerates adhered to the larger surfaces, suggesting a crystalline structure. The morphological analysis confirms the formation of a material with structural characteristics suitable for applications that require high porosity and possibility of functionalization. EDS analysis confirms the presence of zinc in the material structure (Figure 3C).

**Figure 3:**
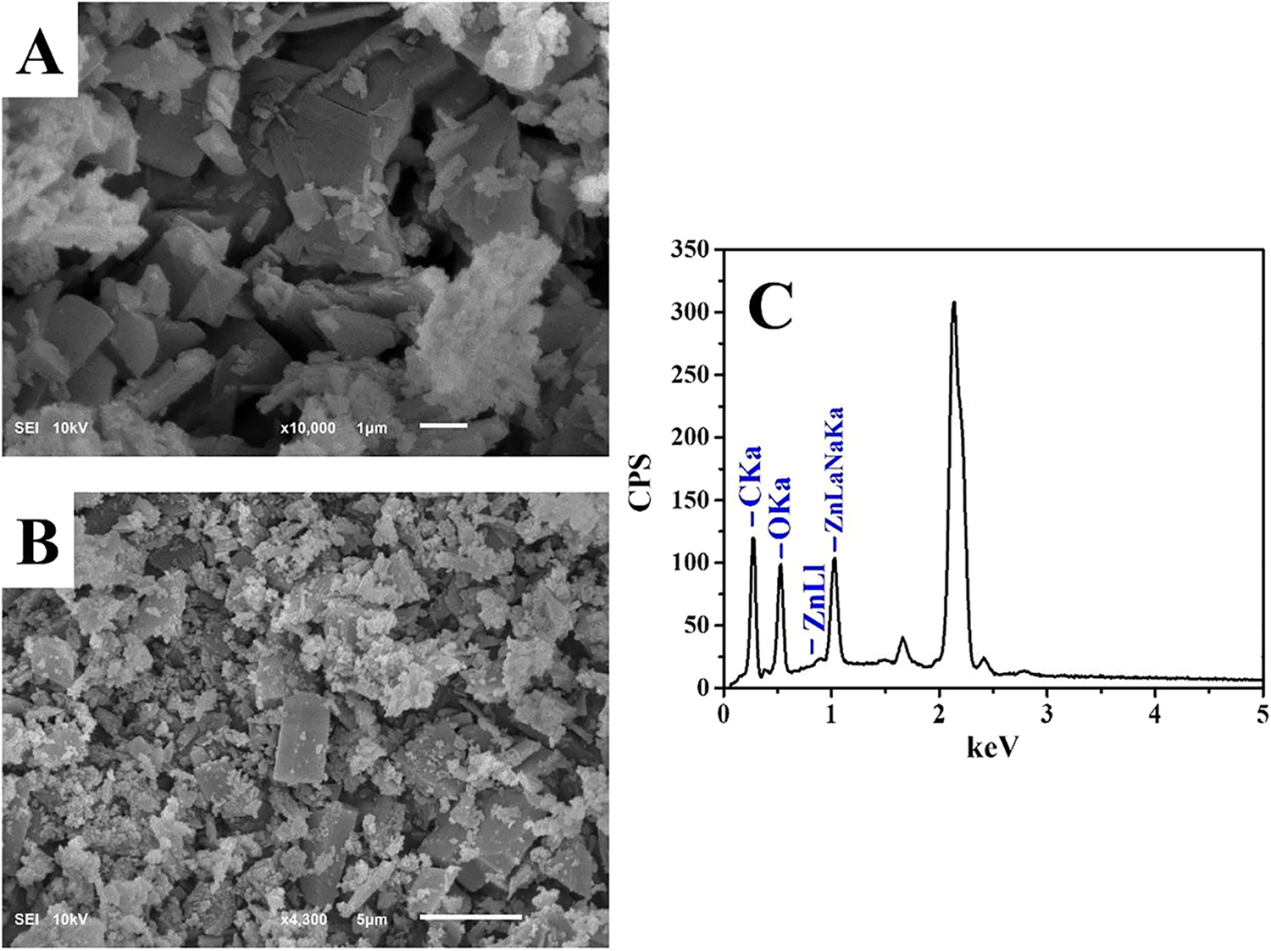
Scanning electron microscopy (SEM) of the bioMOF at magnifications of (A) ×10,000 and (B) ×4,300, showing particles with irregular morphology and heterogeneous distribution. (C) Energy dispersive X-ray spectroscopy (EDS) spectrum of the material, indicating the elements C, O, and Zn.

### 3.2 Loading Efficiency

Encapsulation efficiency is a critical parameter for evaluating the performance of drug delivery systems, as it directly influences drug transport, storage capacity, and release behavior. The Cur content present in the material was quantified by UV/vis absorption spectroscopy. The analytical calibration curve exhibited excellent linearity over the concentration range of 30–200 mg L^–1^, following the equation of Absorbance = 0.00299­[Cur] – 0.0132 and *R* ^2^ of 0.999 (Figure S2). The value found for the Cur concentration was 126.7 ± 3.36 mg L^–1^, representing an encapsulation efficiency of 75.98 ± 2.65% (*n* = 3). Figure S3 shows adsorption/desorption curve of the material. [Zn­(Btc­(COO)_3_)­(Phe)]*_n_* after Cur loading. Following drug loading, the specific surface area decreased substantially to approximately 2.4 m^2^ g^–1^. This value, together with the drug loading results, supports the successful incorporation of the drug within the porous structure of the material. These findings demonstrate that [Zn­(Btc­(COO)_3_)­(Phe)]*_n_* possesses a high loading capacity and suitable structural characteristics for drug encapsulation, highlighting its potential applicability as a carrier system for biomedical and controlled drug delivery applications.

### 3.3 Cell Viability

The biological activity of free Cur (Figure S4A), [Zn­(Btc­(COO)_3_)­(Phe)]*_n_* (Figure S4B) and [Zn­(Btc­(COO)_3_)­(Phe)]*_n_* @Cur complex (Figure S4C) was investigated against the human breast adenocarcinoma cell line (MCF-7) using cell viability assays (Figure S4). Both were tested at concentrations varying from 3.12 to 200 μmol L^–1^. Figure 4A displays the concentration-dependent effect of the complex and the isolated active on cell viability.

**Figure 4:**
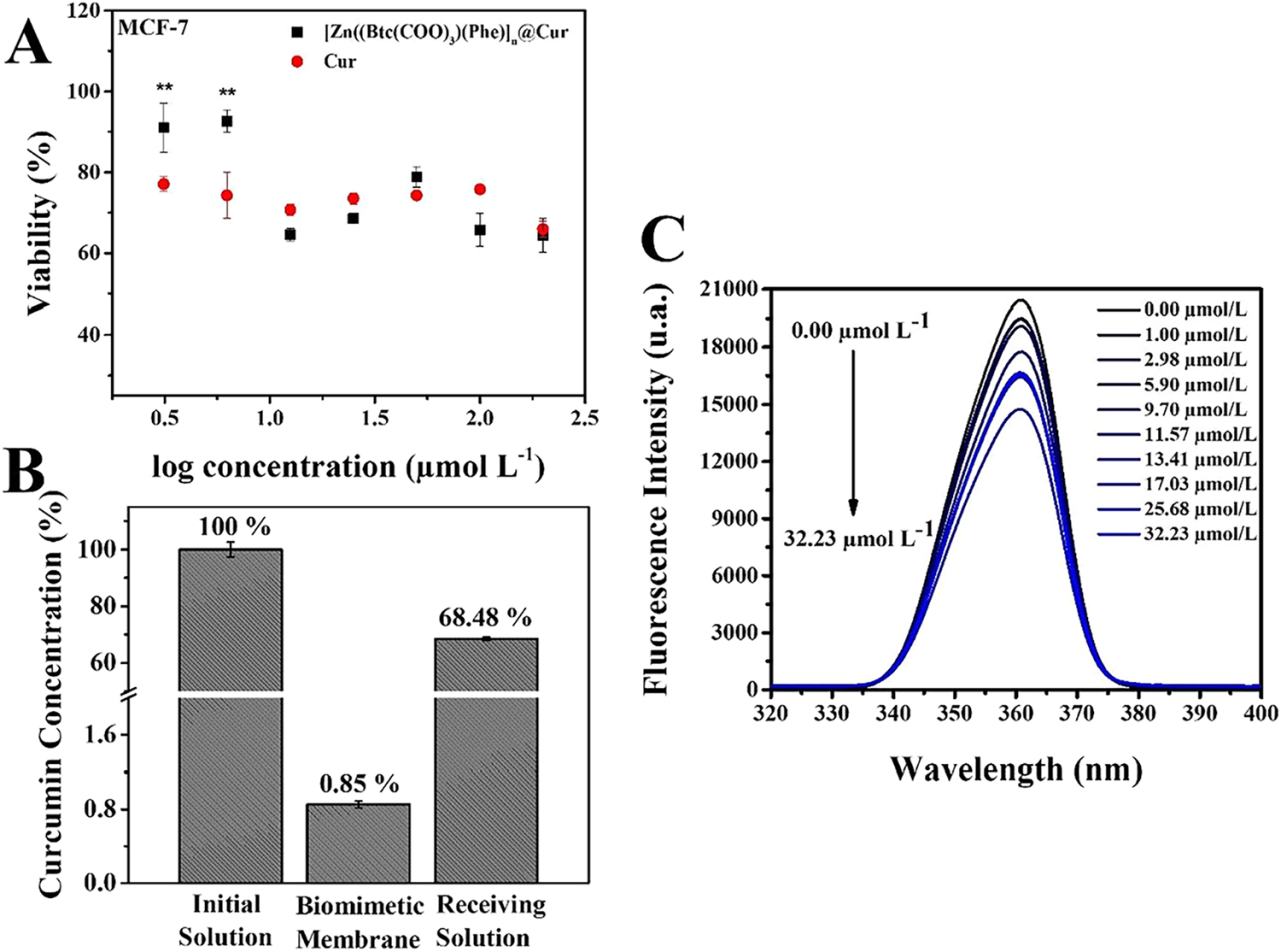
*In vitro* studies of the efficiency of the material produced. (A) Cell viability. Human breast cancer cells (MRC-7) after 48 h of treatment with Cur or [Zn­(Btc­(COO)_3_)­(Phe)]*_n_* @Cur in different concentrations. **p < 0.01 (*n* = 3). (B) Permeation of Cur present in [Zn­(Btc­(COO)­3)­(Phe)]*_n_* @Cur into the skin layers through a biomimetic membrane. (C) Fluorescence spectra of BSA in the presence of the material; λ_exc_ = 280 and λ_emi_ = 350 nm, using concentrations ranging from 0 to 32.23 μmol L^–1^.

Viability was observed to decrease sharply until reaching a plateau at 68%, indicating that the compound exerts a partial inhibitory effect under the tested conditions. Compared to free Cur, the [Zn­(Btc­(COO)_3_)­(Phe)]*_n_* @Cur complex promotes a more pronounced reduction in cell population at concentrations close to 10 μmol L^–1^. Analysis of the empty matrix [Zn­((Btc­(COO)_3_)­(Phe)]*_n_*) (Figures 4A and S4) revealed that the carrier also possesses intrinsic biological activity, reducing cell viability to levels near 65% at high concentrations (200 μmol L^–1^). The greater efficiency of the complex in reducing cell survival compared to free Cur suggests that the framework not only acts as a delivery system but may also potentiate the antitumor action through a synergistic effect between the matrix and the encapsulated drug. The observed cytotoxicity limit (68%) may be related to the saturation of cellular uptake mechanisms or an intrinsic resistance of the MCF-7 line to the mechanism of action of this specific system. This occurs because these cells exhibit properties of self-renewal, quiescence, and overexpression of drug efflux transporters, which are among the factors responsible for chemoresistance in breast cancer patients.^53^

### 3.4 Release Kinetics

The Cur release kinetics were investigated to evaluate the potential of [Zn­(Btc­(COO)_3_)­(Phe)]*_n_* @Cur as a smart biomaterial for controlled drug delivery applications. This analysis provides important information regarding the release behavior of the system over time, which is essential for ensuring therapeutic efficiency and safety in biomedical applications. To simulate physiological conditions, the release experiments were conducted in phosphate-buffered saline (PBS, pH 7.4). The release profile of Cur from the material was monitored over time, enabling a detailed assessment of the amount of drug released at different intervals. Figure S5 presents the release profile, highlighting the kinetic behavior of the system throughout the experiment. To better understand the release mechanism, the experimental data were fitted using two mathematical models, (Higuchi and Ritger-Peppas models). The kinetic parameters obtained from each model are summarized in Table 1.

**Table 1:** Kinetic Study Parameters

model	parameters	value
Higuchi	*K*_H_ (min^1/2^)	0.001
	*R* ^2^	0.934
Ritger-Peppas	*K* (min^–1^)	0.13
	*n*	0.31
	*R* ^2^	0.948

It is observed that the Ritger-Peppas model described the release profile in the studied interval with greater precision, with a coefficient of determination (*R* ^2^) of 0.948. This parameter allows the identification of the predominant drug transport mechanism. Values of *n* less than 0.43 characterize Fickian diffusion, while values between 0.43 and 0.85 correspond to anomalous (non-Fickian) transport, and values greater than 0.85 correspond to type II transport, respectively.^46^ The experimental value obtained was 0.31, suggesting that the release of Cur occurred mainly through a diffusion process. Complementarily, the fit obtained for the Higuchi model (*R* ^2^ = 0.934) corroborates the findings of the Ritger-Peppas model, reinforcing that the resistance to mass transport is located in the nanocarrier matrix. These data suggest that the release system maintains its structural integrity, without suffering immediate degradation or drastic physical changes in the study environment.^54^

### 3.5 Permeation in Biological Barrier

To investigate the permeation ability of Cur across biological barriers, an *in vitro* assay was performed using a biomimetic membrane model designed to simulate physiological permeation conditions. Figure 4B illustrates the levels of Cur in both the biological membrane and the receptor medium after 24 h of exposure. The results demonstrated that the Cur released from [Zn­((Btc­(COO)_3_)­(Phe))]*_n_* @Cur was capable of permeating the membrane and reaching the receptor compartment, indicating the efficiency of the formulation in overcoming biological barriers. From the total Cur concentration applied to the membrane (96.73 mg L^–1^) approximately 0.85% remained retained within the biological membrane, whereas 68.48% was detected in the receptor solution after 24 h. These findings suggest efficient Cur diffusion through the biomimetic barrier and reinforce the potential applicability of the material as a drug delivery platform.

### 3.6 Study of Interaction with Albumin

BSA was selected as the model protein for evaluating the interaction between the nanocarrier and biological macromolecules. BSA exhibits intrinsic fluorescence, primarily associated with tryptophan residues, with a characteristic emission maximum near 360 nm upon excitation at 280 nm. Variations in tryptophan fluorescence intensity, such as quenching or enhancement, may indicate conformational changes in the protein structure and suggest interactions between BSA and the nanocarrier system. The fluorescence results are presented in Figure 4C. Under the experimental conditions employed, BSA displayed a broad and intense emission band centered at approximately 360 nm when excited at 280 nm. Upon successive additions of aliquots (5.0 μL) of the [Zn­((Btc­(COO)_3_)­(Phe))]*_n_* @Cur suspension (401 μmol L^–1^), a gradual decrease in fluorescence intensity was observed as the nanocarrier concentration increased from 0 to 32.23 μmol L^–1^. This progressive fluorescence quenching suggests an interaction between BSA and the bioMOF system, possibly associated with binding events and alterations in the local environment of tryptophan residues.

Fluorescence quenching is a process in which the fluorescence intensity of a fluorophore decreases due to the presence of a quenching agent, typically as a result of molecular interactions or energy transfer processes.^55^ Analysis of the data using the Stern–Volmer equation revealed an approximately linear behavior (Figure S6), resulting in a constant *K*_SV_ of 9.4 × 10^3^ M^–1^. Adamczyk et al.^56^ report high-thickness interactions for a stable drug–protein complex, for the BSA-5FU system (*K*_SV_ = 3.2 × 10^4^ M^–1^). The moderate interaction profile observed experimentally in this work suggests a more reversible binding. This characteristic is potentially advantageous for facilitating drug release at the target site, avoiding permanent retention by plasma proteins. However, this moderate affinity implies a dynamic balance between the nanocarrier and the biological medium. Although the interaction may facilitate transport and favor circulation time, the intermediate binding strength also points to a risk of premature leakage or displacement by endogenous competitors. Small deviations from linearity at higher concentrations indicate the possible presence of multiple interaction sites or combined quenching mechanisms. These results suggest a propensity for interaction between the synthesized material and BSA.

## 4 Conclusion

In conclusion, this study demonstrated the successful synthesis and characterization of bioMOF for the controlled release of Cur. It provides fundamental insights into the development of bio-MOF-based systems for the delivery of hydrophobic compounds. Characterization of [Zn­(Btc­(COO)_3_)­(Phe)]*_n_* @Cur structure confirmed its structural stability and its ability to incorporate curcumin. Kinetic data followed best fit for the Ritger-Peppas model, offering a preliminary understanding of how these matrices control drug release under physiological conditions. Furthermore, the moderate interaction with BSA suggests that, although the nanocarrier can interact with transporter proteins, further studies are needed to fully elucidate its behavior in complex biological media. While the complex demonstrated enhanced efficacy against MCF-7 cells compared to free Cur at specific concentrations, the observed viability plateau of 68% highlights the challenges to the overall efficacy of the drug in resistant cell lines. Thus, these results contribute to the mechanistic understanding of MOFs as delivery platforms, serving as a basis for the future development of more sophisticated and targeted phytochemical carriers.

## Supplementary Material



## Notes

t-plot constructed using the Harkins-Jura thickness equation for the pristine MOF; analytical curve of curcumin in the concentration range of 30 to 200 mg L^–1^ for loading efficiency determination; N_2_ adsorption/desorption curve of [Zn­(Btc­(COO)_3_)­(Phe)]_*n*_ after Cur loading; release kinetics of Cur present in [Zn­(Btc­(COO)_3_)­(Phe)]*_n_* @Cur through a cellulose dialysis membrane; Stern-Volmer plot for the fluorescence quenching of Bovine Serum Albumin­(BSA) by the [Zn­(Btc­(COO)_3_)­(Phe)]*_n_* @Cur nanocarrier (PDF)

## Abbreviations

BET: Brunauer–Emmett–Teller method
bio-MOF: biocompatible metal–organic frameworks
BSA: bovine serum albumin
Btc­(COOH)_3_: benzene-1,3,5-tricarboxylic acid
Cur: curcumin
DMSO: dimethyl sulfoxide
FT-IR: fourier-transform infrared spectroscopy
K_SV_: Stern–Volmer constant
MOF: metal–organic frameworks
MTT: 3-(4,5-dimethylthiazol-2-yl)-2,5-diphenyltetrazolium bromide
PBS: phosphate-buffered saline
Phe: l-phenylalanine
P-XRD: powder X-ray diffraction
SEM-EDS: scanning electron microscopy with energy-dispersive X-ray spectroscopy
TG/dTG: thermogravimetric analysis/derivative thermogravimetry
UV–vis: ultraviolet–visible spectroscopy
Zn: zinc
