# Supplementary material for: Zinc-Based bioMOF Nanocarrier for Curcumin Release Enables Permeation through Biomimetic Biological Membranes and Controlled Release
Source: ACS Omega. 2026 Jul 17;11(30):44729–38. doi: 10.1021/acsomega.6c00840 (PMC13448945; doi:10.1021/acsomega.6c00840)
Supplement: Supplementary file 1 [file ao6c00840_si_001.pdf]

Supplementary Material

Zinc-based bioMOF nanocarrier for curcumin release enables permeation through biomimetic biological membranes and controlled release

Lorrayne Ohana Coelho[a,b], Marcos Vinicius de Sousa Pereira[a,b], Tatianny de Araujo Andrade[a,b], Katia M. Oliveira[c], Jemmyson R. de Jesus[a,b*]

[a]Research Laboratory in bionanomaterials, LPbio, Department of Chemistry, Federal University of Viçosa, 36570-900 Viçosa, Minas Gerais, Brazil.

[b]National Institute of Science and Technology of Bioanalytics-Lauro Kubota (INCTBio-LK), Campinas, SP 13083970, Brazil

[c]University of Brasilia, Institute of Chemistry, CEP 70910-000, Brasília-DF, Brazil

*Corresponding authors:

Prof. Jemmyson R. de Jesus (jemmyson.jesus@ufv.br)

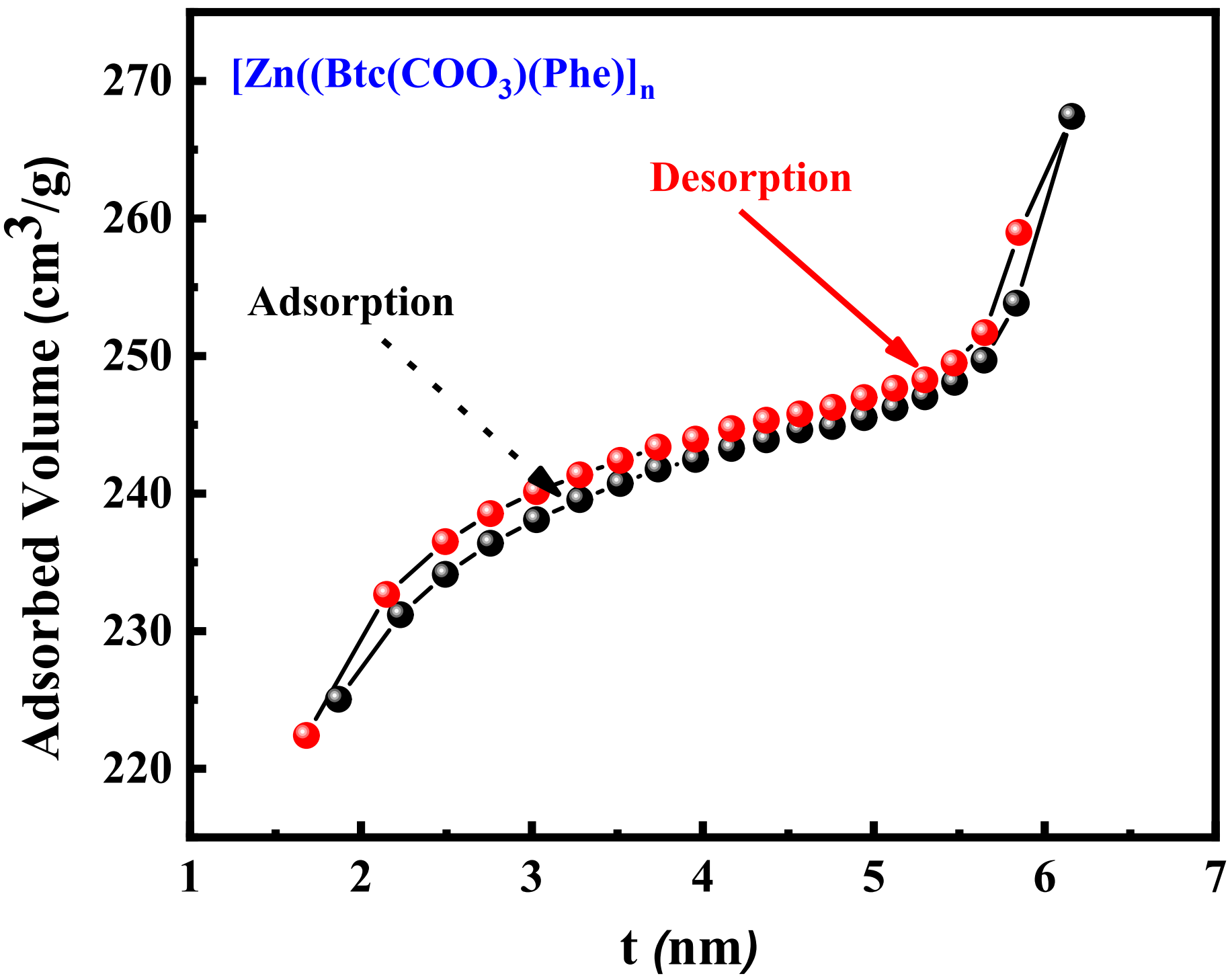

Figure S.1. t-plot constructed using the Harkins-Jura thickness equation for the pristine MOF. The linear region ($0.35 < P/P_0 < 0.80$) was used to estimate the external surface area and micropore volume. The intercept indicates a significant micropore contribution, confirming the predominantly microporous nature of the material.

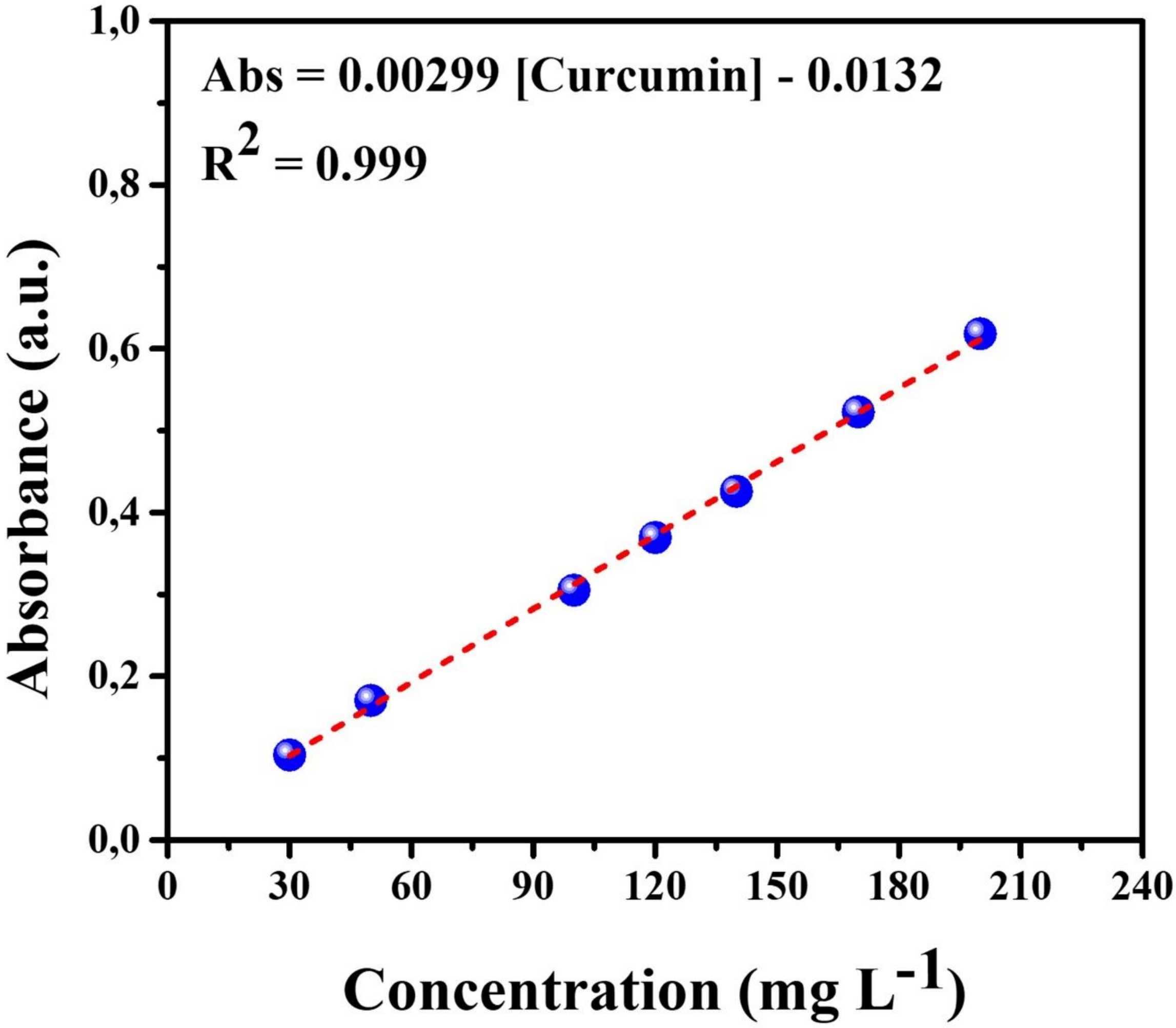

Figure S.2. Analytical curve of curcumin in the concentration range of 30 to 200 mg L^{-1} for loading efficiency determination. The plot displays the absorbance at λ_{max} = 426 nm as a function of concentration, featuring the linear regression equation (Abs = 0.00299 [Curcumin] + 0.0132) and the coefficient of determination (R^2 = 0.999).

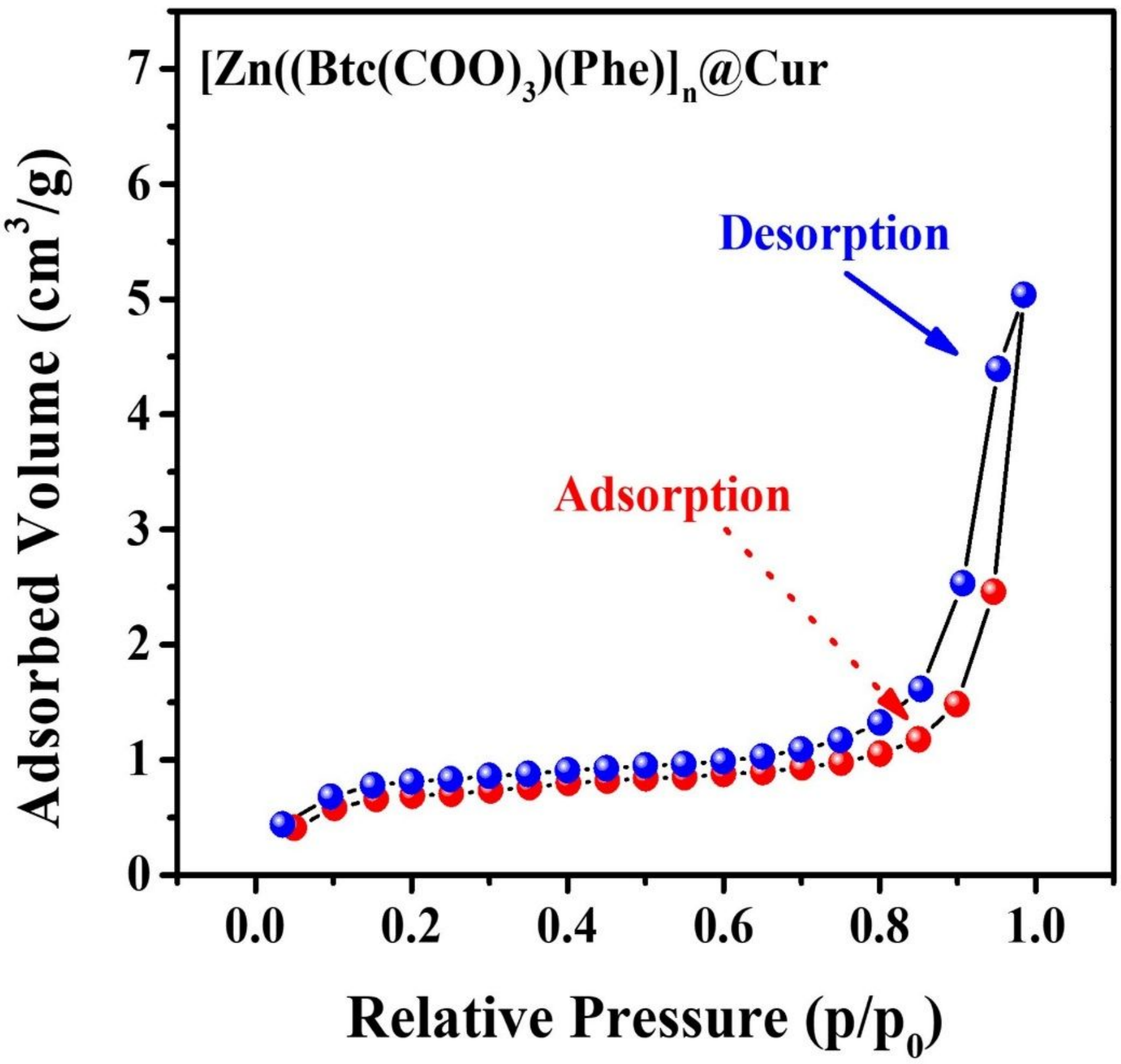

Figure S3. N_2 adsorption/desorption curve of [Zn(Btc(COO)$_3$)(Phe)]ₙ after Cur loading.

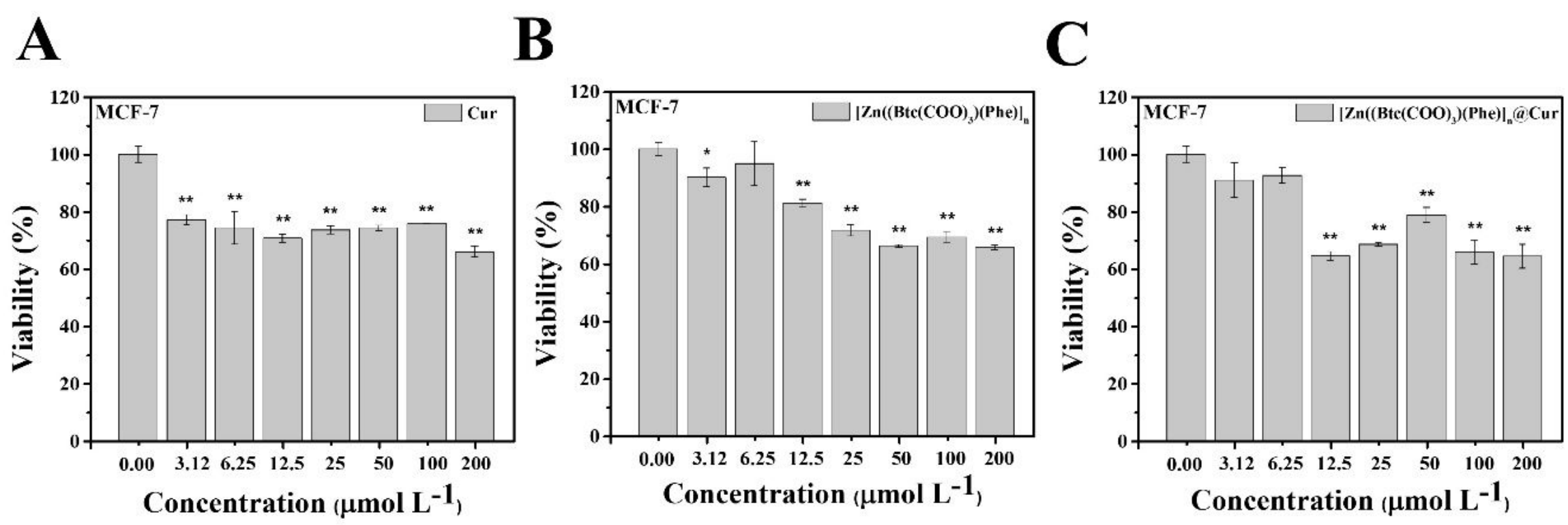

Figure S.4. Cell viability of MCF-7 cells after exposure to different concentrations (0-200 μmol L^{-1}) of **(A)** curcumin (Cur), **(B)** [Zn(Btc(COO)$_3$)(Phe)]ₙ, and **(C)** [Zn(Btc(COO)$_3$)(Phe)]ₙ@Cur. Cell viability was evaluated by the MTT assay and expressed as percentage relative to the untreated control (0 μmol L^{-1}). Data are presented as mean ± standard deviation (SD). Statistical significance compared to the control group is indicated by $^*p < 0.05$ and $^{**}p < 0.01$.

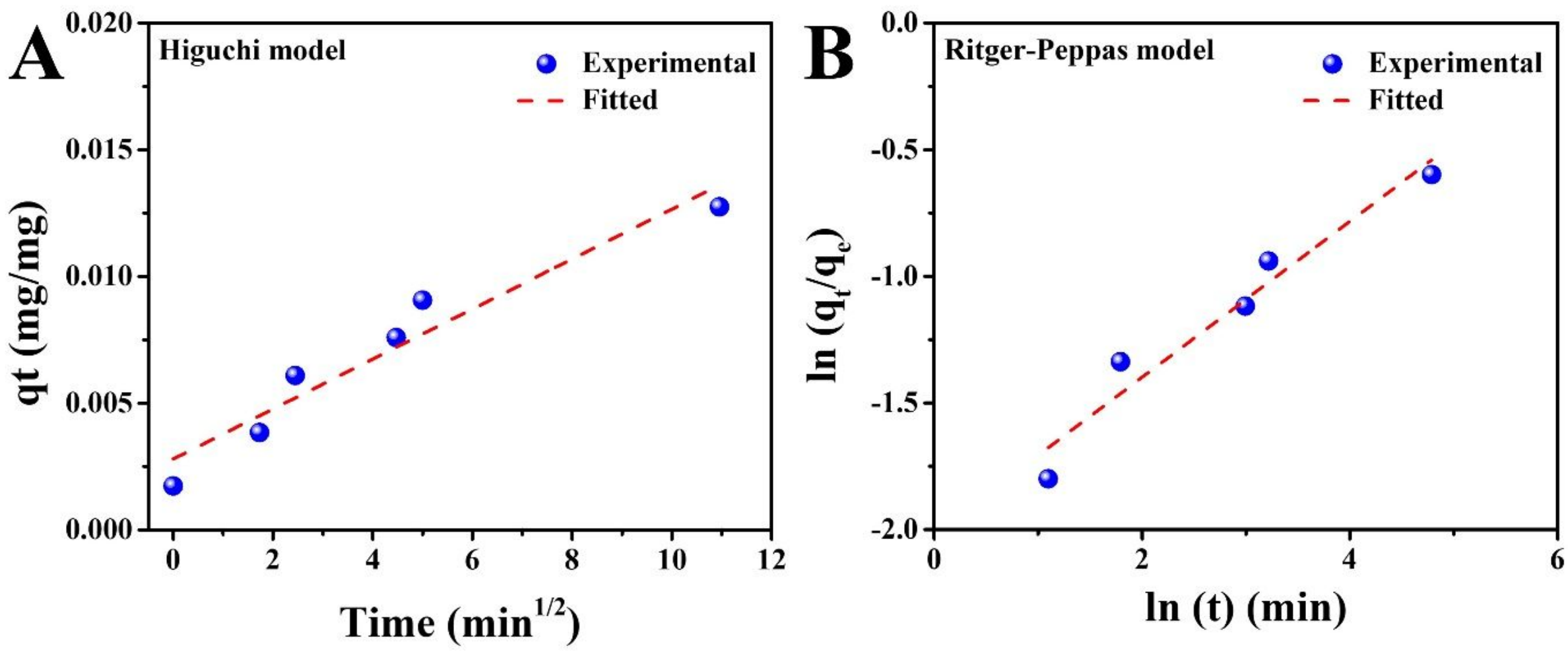

Figure S.5. Release kinetics of Cur present in $[Zn((Btc(COO)_3)(Phe)]_n$@Cur through a cellulose dialysis membrane. (A) Higuchi model and (B) Ritger-Peppas model.

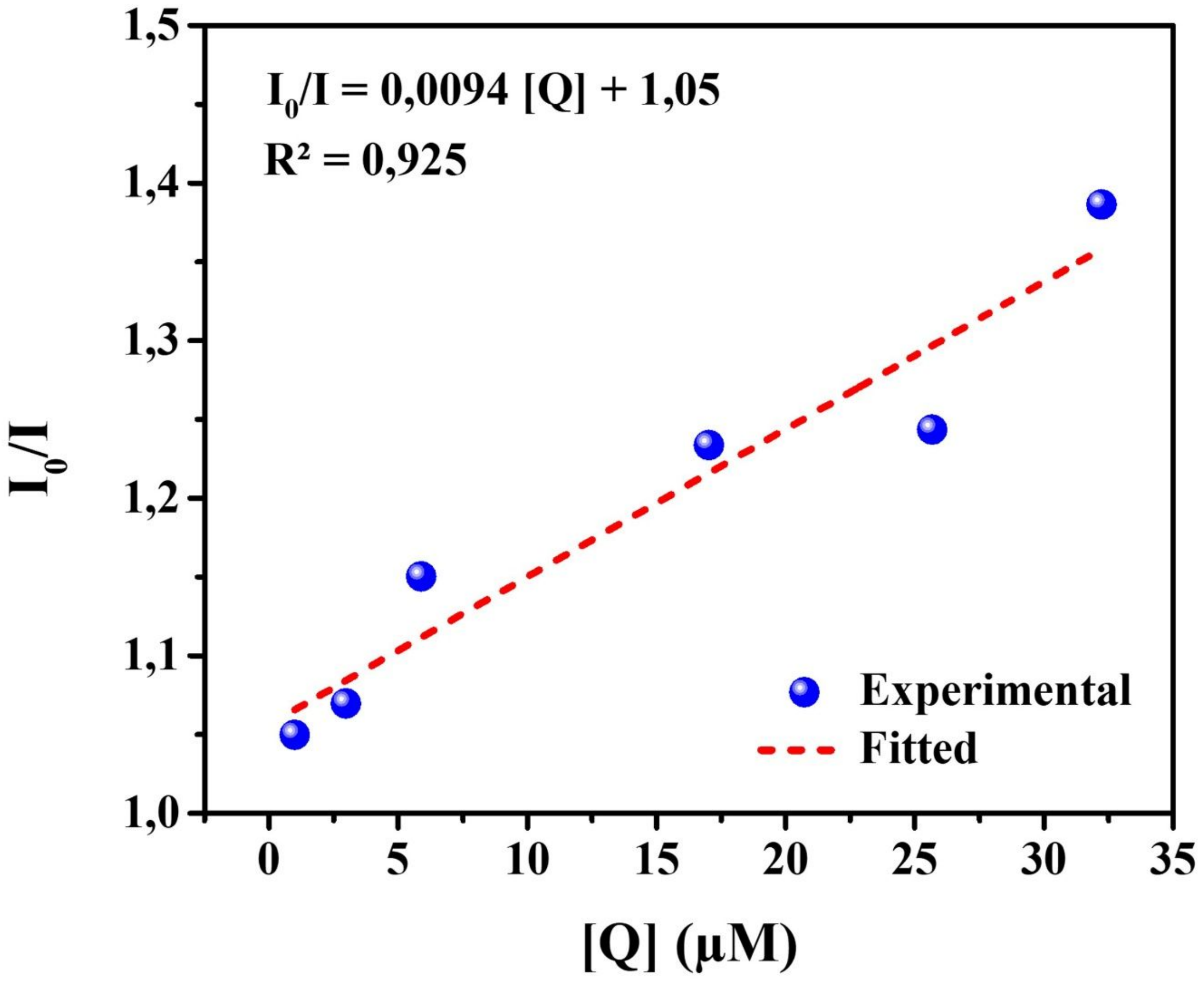

Figure S.6. Stern-Volmer plot for the fluorescence quenching of Bovine Serum Albumin (BSA) by the $[Zn((Btc(COO)_3)(Phe)]_n$@Cur nanocarrier. The plot illustrates the ratio of fluorescence intensities (I_0/I) as a function of the quencher concentration ([Q] in µM). The linear regression ($R^2 = 0.925$) provides the Stern–Volmer constant ($K_{SV} = 9.4\times10^3$ M^{-1}), indicating a moderate binding affinity.
